# Phylogenetic Tree Inference: A Top-Down Approach to Track Tumor Evolution

**DOI:** 10.3389/fgene.2019.01371

**Published:** 2020-02-07

**Authors:** Pin Wu, Linjun Hou, Yingdong Zhang, Liye Zhang

**Affiliations:** ^1^School of Life Science and Technology, ShanghaiTech University, Shanghai, China; ^2^University of Chinese Academy of Sciences, Beijing, China; ^3^Library and Information Center, ShanghaiTech University, Shanghai, China

**Keywords:** phylogenetics, tumor evolution, multi-region sequencing, lineage tracing, allele frequency

## Abstract

Recently, an increasing number of studies sequence multiple biopsies of primary tumors, and even paired metastatic tumors to understand heterogeneity and the evolutionary trajectory of cancer progression. Although several algorithms are available to infer the phylogeny, most tools rely on accurate measurements of mutation allele frequencies from deep sequencing, which is often hard to achieve for clinical samples (especially FFPE samples). In this study, we present a novel and easy-to-use method, PTI (Phylogenetic Tree Inference), which use an iterative top-down approach to infer the phylogenetic tree structure of multiple tumor biopsies from same patient using just the presence or absence of somatic mutations without their allele frequencies. Therefore PTI can be used in a wide range of cases even when allele frequency data is not available. Comparison with existing state-of-the-art methods, such as LICHeE, Treeomics, and BAMSE, shows that PTI achieves similar or slightly better performance within a short run time. Moreover, this method is generally applicable to infer phylogeny for any other data sets (such as epigenetics) with a similar zero and one feature-by-sample matrix.

## Introduction

Cancer is an evolutionary process that is shaped by selection pressure and the accumulation of somatic mutations, resulting in a high level of heterogeneity within and between tumor samples (Marusyk et al., 2012; Yates and Campbell, 2012). Such heterogeneity in genomes can be used to distinguish tumor subclonal populations and track the evolutionary trajectory of cancer progression. Metastasis is normally considered as the last step during cancer progression and is still the major cause of cancer death but poorly understood mechanistically. A number of studies sequenced multiple biopsies of primary and metastatic tumors to elucidate the order of mutation accumulation and the origin of distal metastasis (Gundem et al., 2015; Yates et al., 2017; Ferronika et al., 2019). A better understanding of metastasis process may eventually lead to novel diagnosis and treatment strategies.

A number of computational methods are available to infer the genotypes of tumor cell populations. However, most existing methods infer the phylogeny of cancer evolution based on somatic mutations variant allele frequencies (VAFs) from DNA deep sequencing data (Jiao et al., 2014; Malikic et al., 2015; Yates et al., 2015; Nieboer et al., 2018). Several traditional phylogenetic inference methods utilize multiple sequence alignments, neighbor joining with Pearson correlation distances, maximum parsimony algorithm or maximum likelihood algorithm based on variant presence patterns across samples (Kim et al., 2015; Lu et al., 2016; Zhao et al., 2016; Choi et al., 2017; Naxerova et al., 2017; Zhai et al., 2017). Most of these methods are computationally intensive and require a long running time. In 2015, LICHeE was developed to construct multi-sample tumor phylogenetic trees and tumor subclonal decomposition from accurate VAFs of somatic single nucleotide variants (SSNVs) obtained by deep sequencing (Popic et al., 2015). LICHeE first groups subsets of somatic mutations that have similar presence-absence patterns as well as similar VAFs across multiple tumor samples. Then, it constructs a constrained network to infer the relationships among clusters of somatic mutations and identify tumor phylogenetic trees. Several other methods adopt similar principles but different methodological frameworks, such as Treeomics and BAMSE (Reiter et al., 2017; Toosi et al., 2019). Treeomics was developed to reconstruct the phylogeny of metastases and map subclones to their anatomic locations. It uses total reads and variant reads of SSNVs from multiple related normal and tumor samples of individual cancer patients as input files. Then it uses a Bayesian inference model to identify evolutionarily compatible mutation patterns and then infer evolutionary trees. Another probabilistic method, named BAMSE infers subclonal history and lineage tree reconstruction of heterogeneous tumor samples using somatic mutations read counts as input. The posterior probability of tree is inferred by a Bayesian model that integrates prior belief about the number of subclones, the composition of the tumor, and the process of subclonal evolution. However, users have to decide the number of subclones, which is normally difficult to estimate. There are two major issues common for these methods. Most importantly, it is often difficult to obtain accurate allele frequency from clinical samples, such as formalin-fixed, paraffin-embedded (FFPE) samples (Astolfi et al., 2015). Results of these methods are also sensitive to several key parameters, and yet there is no easy way for users to decide on these parameters.

Here, we propose PTI (Phylogenetic Tree Inference), a novel method which use an iterative top-down approach to infer the phylogenetic tree structure of multiple tumor biopsies from same patient using somatic mutations without the needs of accurate allele frequencies. In addition, PTI has only one parameter to set, and we also provide clear instructions on how to set this parameter.

## Methods

**Figure 1:**
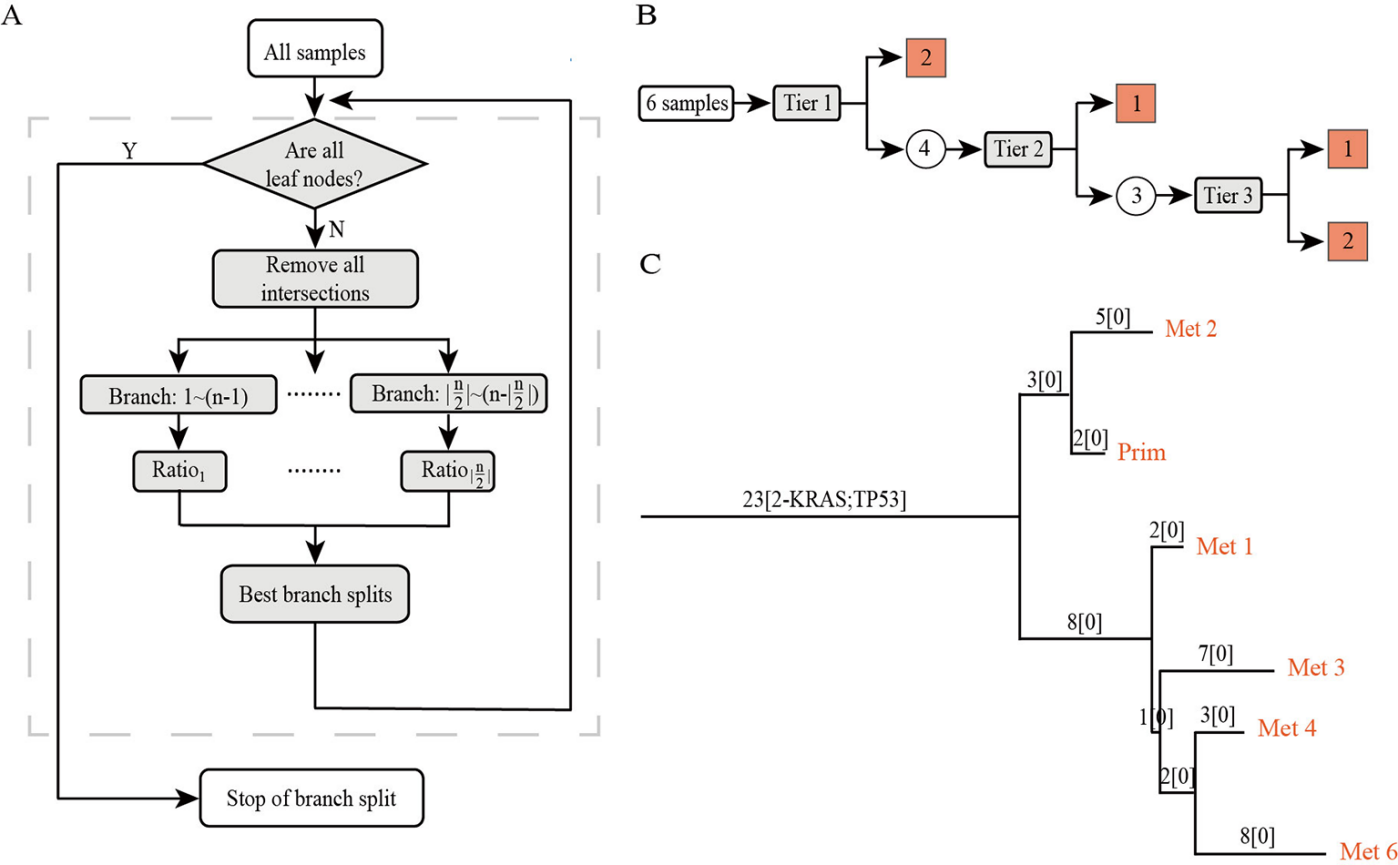
The overview of PTI. **(A)** The workflow of PTI. **(B)** Taking the patient with six samples as an example, the specific process of PTI inferring the phylogenetic tree is shown in detail. The square grid indicates that the leaf node has been reached, and the circle indicates that the remaining samples still have to be iterated to find the best branch split. **(C)** An example of a phylogenetic tree inferred by PTI. The phylogenetic tree is a rooted tree whose leaf nodes are samples. The annotation information on the branches includes the length of the branches, which is equal to the number of shared mutations, and the annotation of driver genes.

PTI is a method designed to use an iterative top-down approach to infer the rooted phylogenetic tree among multiple samples of the same patient. In this section, we provide an overview of our approach (**Figure 1**). First, PTI identifies shared mutations for all samples and defines the number of shared mutations as the length of the root trunk. Then, PTI uses an iterative top-down approach to find the optimal branch split until all samples reach the leaf nodes. PTI also annotates the mutations of known driver genes on the tree structure, which facilitates an intuitive understanding of the key mutation events during cancer progression.

### Identify and Remove the Shared Mutations From All Samples

Assume we obtained multiple samples *s*, *s* ∈ {1,2,…,*n*} from same patient. Using somatic mutation caller, such as Mutect2 or VarScan2 (Koboldt et al., 2012; Cibulskis et al., 2013), we can detect the mutation allele frequencies in each sample. Define these somatic mutations by *r*_1_,*r*_2_…,*r_m_*. We then build a binary matrix *M* with rows labeled *r*_1_,*r*_2_…,*r_m_*, and columns labeled *s*_1_,*s*_2_…,*s_n_*, such that *M_ij_* = 1 if and only if the VAF of somatic mutation *r_i_* in sample *s_j_* is greater or equal to a given threshold. The more high-confidence mutations are used to construct a phylogenetic tree, the more accurate the structure of the tree. However, when the number of somatic mutations within a patient is too large, an optional filtering step of somatic mutations based on allele frequency (default is 0.1) can be implemented by PTI. Matrix *M* is the input of PTI. We calculate the intersection of mutations in all samples of the same patient and define the intersection as the length of the root trunk. After removing the shared mutations from all samples, the next step is to find the optimal branch split for the filtered data set *M_filter_*.

### Find the Optimal Branch Split

Genomic variations in cancer cells gradually accumulate over the course of carcinogenesis and cancer development (Gerlinger et al., 2012; Sato et al., 2016). So despite the complexity in cancer evolution, more than two ways split is rarely observed on the evolutionary tree in existing studies (Hong et al., 2015; Schwarz et al., 2015; Brown et al., 2017). Therefore, PTI will iterate through all possible two-way branch splits, $S=\left\{\left(1,n-1\right),\left(2,n-2\right),\ldots \left(\left|\frac{n}{2}\left|,n-\right|\frac{n}{2}\right|\right)\right\}$ to infer the optimal branch split. Notably, our method indeed is able to detect more than two ways split at any given evolutionary node (**Supplementary Note 1**).

For each possible branch split *S_t_* **,** $t\in \left\{1,2,\ldots ,\left|\frac{n}{2}\right|\right\}\;,{∁}_{n}^{t}$ combinations are included. Let *θ* be an object of the numbers of shared mutations measured on all possible branch splits. For each possible branch split *S_t_* and combination *c*, $c\in \left\{1,2,\ldots ,{∁}_{n}^{t}\right\}$the corresponding element *θ_tc_*, represents the number of shared mutations in the larger group. If *n* is even, there is no larger group of the possible branch split $\left(\frac{n}{2},n-\frac{n}{2}\right)$. Then in this case, $\theta_{\frac{n}{2}c}\;$presenting the smaller number of shared mutations in two equal size groups.

In order to determine which possible branch split is the best, we define ∂ to be an vector of ratio measured on all possible branch splits which is calculated via the Equation (1), where *θ*_t__max_ represents the maximum value and *θ*_t__sec_max_ represents the secondary maximum value in *θ_t_*:

(1) $$\begin{array}{c} \partial_{t}=\frac{\theta_{\text{t max}}}{\theta_{\text{t sec}\_\text{max}}} \end{array}$$

If the optimal split occurs in *S_t_*, then the ratio between the best combination and second best combination should be much larger compared with non-optimal splits (see **Supplementary Note 2** for details description of rationale of using this ratio).

Then, the samples that reach the leaf node after the optimal split will be removed from the data set *M_filter_*. This method will iterate over the rest of the samples until all samples are split into leaf nodes. For a patient, there may be more than one tree structures with an equal ∂ value. To determine the optimal tree structure, an aggregated mutation count (*W_T_*) is calculated for each tree structure using mutations on all trunks that contain two or more leaf nodes. The tree with the largest scores will be the optimal tree. Let *i*, *i* ∈ {0,1,…,*k*}, represents all trunk levels in each tree structure. In trunk level *i*, there are *N_i_* trunks so that we let *j*, *j* ∈ {1, … , *N_i_*}, represent all trunks in each trunk level. We also define *ω_ij_* as the length of trunk *j* in trunk level *i* and define *χ_ij_* which represents the number of leaf nodes involved in trunk *j* in trunk level *i*. Then, the weight score *W_T_* of tree structure *T* will be calculated by:

(2) $$\begin{array}{c} W_{T}=\sum_{i=1}^{k}\sum_{j=1}^{N_{i}}\omega_{ij}\chi_{ij}\; \end{array}$$

without root trunk level (*i* = 0) because the tree structures of same patient have same root trunk.

### Annotation of Driver Mutations on Phylogenetic Tree

It is well known that there are more passenger mutations than driver mutations in cancer genome. Understanding the time of occurrence and the distribution of driver mutations in different samples is important to understand the evolution of tumor progression. Therefore, our method also annotates the putative 299 driver genes on the branches of the tree for downstream analysis (Bailey et al., 2018). It should be noted that in the tree structure, there may be more than one tree branch with annotation information of the same driver gene. This may be caused by the same mutation or different mutations of the same driver gene, which can be answered by an auxiliary information file corresponding to the tree structure file.

As this method assume there is a major single clone for each sample due to multiple biopsies, we do notice that in rare cases this method will output multiple solutions instead of one optimal solution when some samples are consisted of more than one major clones (**Supplementary Note 3**).

## Results

### Results on High-Grade Serous Ovarian Cancer

**Figure 2:**
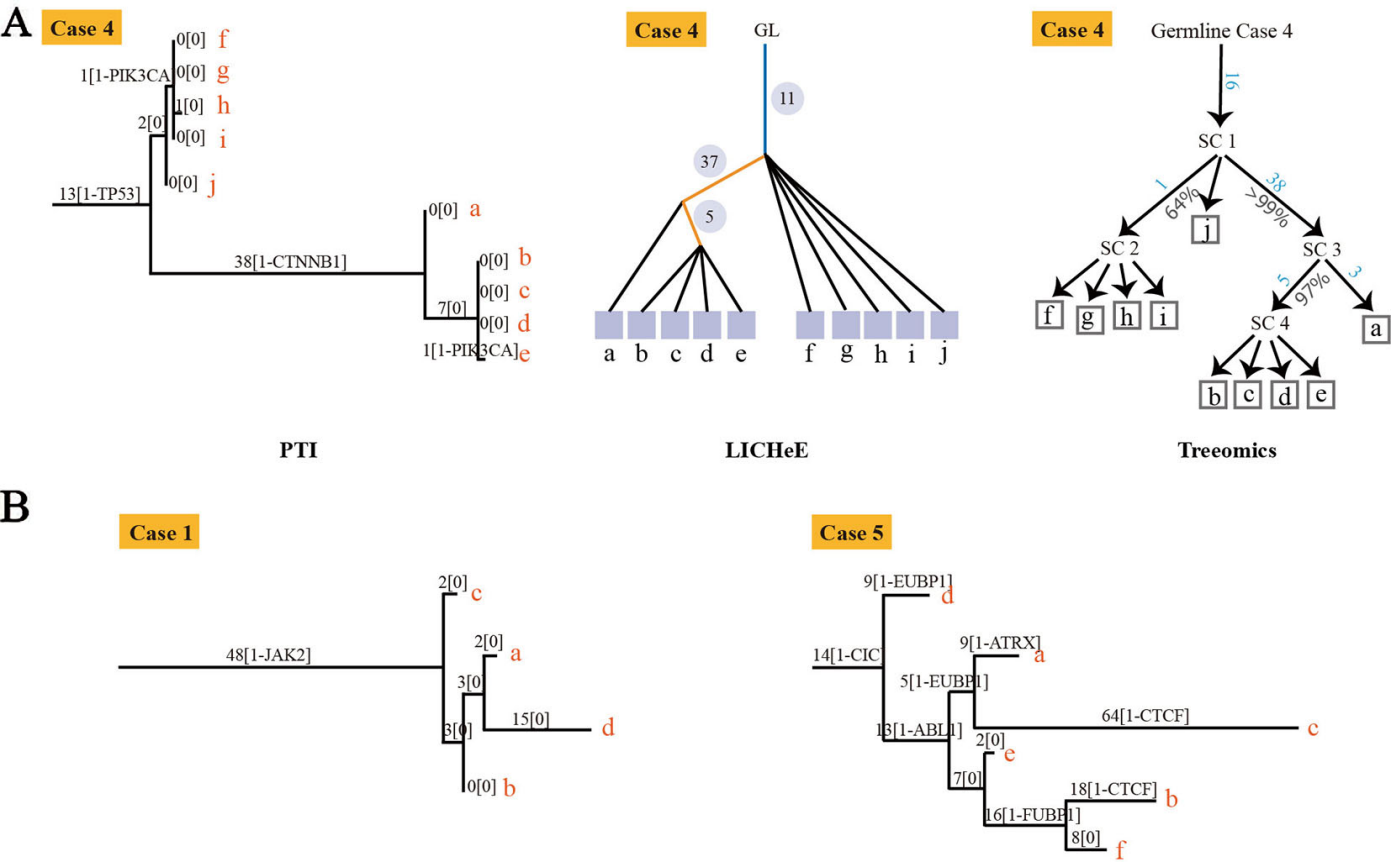
The tree structures inferred by PTI for HGSC Cases 1, 4 and 5. **(A)** The tree structure of Case 4 inferred by PTI, LICHeE, and Treeomics respectively. In the LICHeE result: yellow line, internal branch; light blue line, trunk; black line, contribution link; the numbers inside the circle, SSNVs; the light purple square, tumor region. In the Treeomics result: numbers in blue correspond to the acquired variants in the branches. Percentages (gray) denote bootstrap values (1,000 samples). SC, subclone. **(B)** For Case 1, PTI first separates the sample c to achieve the best branch split, which is same as the result of LICHeE. For Case 5, the tree structure inferred by PTI and Treeomics has high similarities using six samples and eight samples (**Supplementary Note 5**).

To evaluate our method, we compared the performance of PTI with two state-of-the-arts methods, LICHeE and Treeomics on high-grade serous ovarian cancer (HGSC) data set which were obtained from European Genome-Phenome Archive (accession EGAS00001000547) (Bashashati et al., 2013). PTI used all mutations with AF >= 0.01 while LICHeE and Treeomics were run with the parameter defined in their published paper. Then we compared the results of these three methods with the results given in the original literature based on mutations and copy number alterations. In order to evaluate the similarity of two tree structures, we defined a tree structure similarity scoring system. The similarity score represents the proportion of the identical paths in the tree topology and ranges from zero to one (**Supplementary Note 4**). PTI showed slightly better performance compared with LICHeE and Treeomics. Only PTI correctly predicted identical structure in case 4, where sample j and f-i are grouped into one branch (**Table 1** and **Figure 2A**). 4 of 6 results predicted by PTI showed the identical structure which similarity score is equal to 1. None of the three methods showed highly consistent structures in Case 1 and 5 as original results (**Table 1** and **Figure 2B**). In Case 1, results from PTI and LICHeE were highly consistent. For Case 5, when PTI use all somatic mutations with AF >= 0.01, there are only minor differences between the tree structures inferred by PTI and Treeomics. However, if PTI use all single nucleotide somatic mutations obtained from original paper including 8 tumor samples of Case 5, in contrast to the results in the original literature which suggested an early divergence of sample c, PTI first separated the sample h to achieve the most common mutations (n = 4) in the remaining samples (**Supplementary Note 5**). Careful analysis of the somatic mutation data set revealed that if the sample c is diverged first, there is only one shared somatic mutation in the remaining samples.

**Table 1:** The comparison of PTI, LICHeE, and Treeomics based on HGSC data set.

Patient_ID	PTI (AF >= 0.01)	LICHeE	Treeomics
Case 1	0.14	0.15	0.43
Case 2	1	1	1
Case 3	1	1	1
Case 4	1	0.57	0.83
Case 5	0.27	0.23	0.34
Case 6	1	1	1

### Results on Clear Cell Renal Carcinomas Data Set

We performed a separate comparison between PTI, LICHeE, and BAMSE on clear cell renal carcinomas (ccRCC) data set from eight individuals which were obtained from European Genome-Phenome Archive (accession EGAS00001000667) (Gerlinger et al., 2014). Since LICHeE only used the variant allele frequency of somatic single nucleotide variants to reconstruct the phylogenetic process, PTI took the same set of SNVs with AF >= 0.01 as the input data set. Since this data set lacked information about total reads and variant reads of mutation (Treeomics needs such information), so we only compared results of PTI, LICHeE, BAMSE with those from original literature that used VAF-based clustering, variant presence pattern and maximum parsimony algorithm (Gerlinger et al., 2014). It should be noted that the trees inferred by BAMSE were obtained from Toosi et al. (2019). The comparison shows that PTI and LICHeE performed similarly in terms of accuracy and speed in the ccRCC data set while all tree structures inferred by BAMSE had single-branch or multi-branch differences (**Table 2**, **Figure S7**).

**Table 2:** The comparison of PTI and other methods on ccRCC data set.

Patient_ID	PTI (AF >= 0.01)	LICHeE	BAMSE
EV003	0.67	0.48	/
EV005	1	0.70	0.24
EV006	0.89	0.17	0.1
EV007	0.86	0.85	0.33
RMH002	0.55	1	0.55
RMH004	0.5	0.57	0.5
RMH008	1	1	0.83
RK26	0.65	0.54	/

### Results on Breast Cancer Data Set

**Figure 3:**
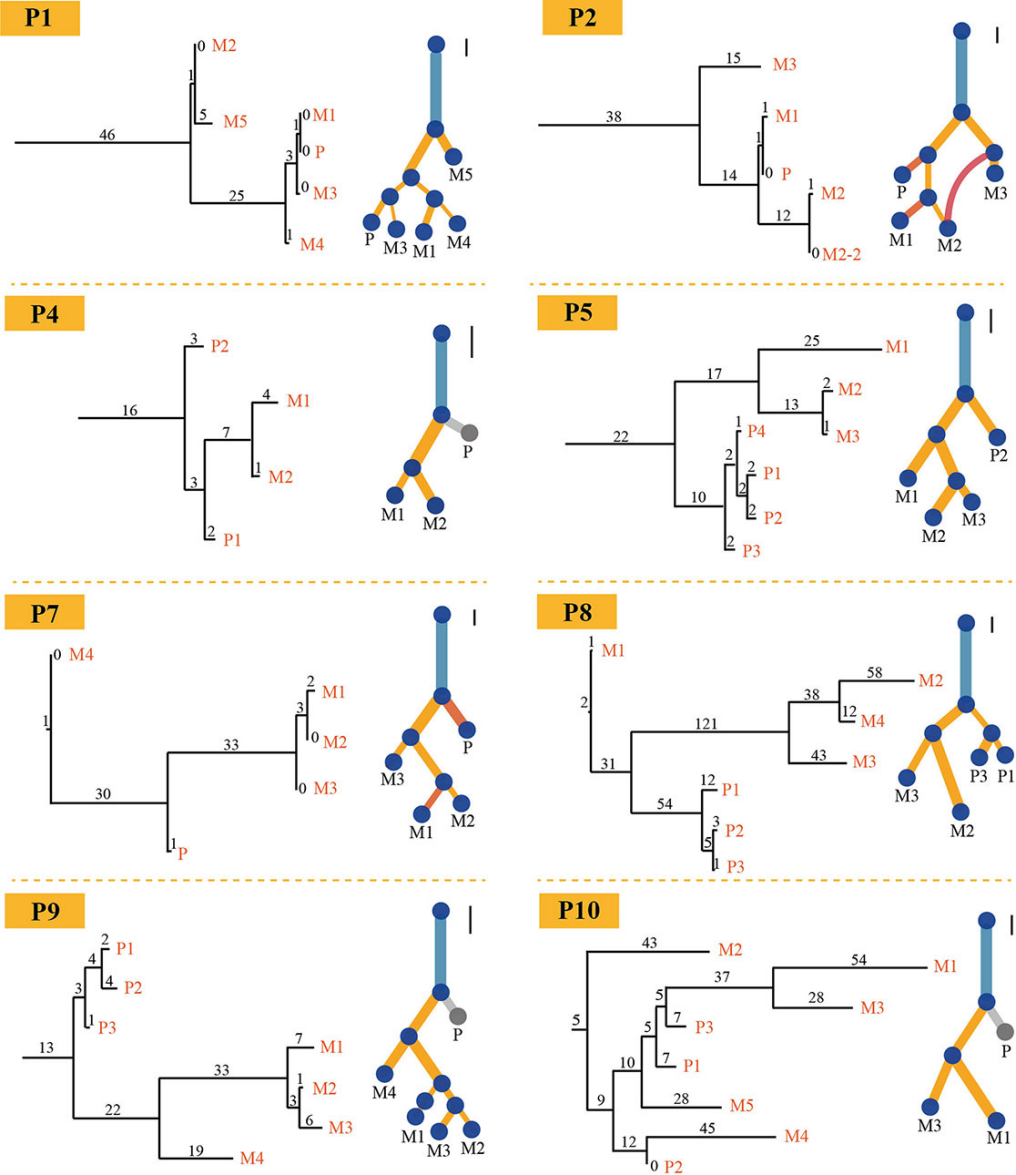
Comparison of trees for eight breast cancer patients. Eight phylogenetic tree structures without annotation information about the driver gene inferred by PTI using all somatic mutations with >=1500X coverage and >=3% VAF (on the left) are compared with the trees (on the right) published in Brown et al. (2017) for each patient on breast cancer data set. As for PTI results, the number of shared mutations is proportional to the length of branch and labeled above each branch. Also, the scale bars on the upper right corner of the results given in original published paper represent 10 SNVs and provide an indication of the original length of the trees.

PTI also was benchmarked with LICHeE and Treeomics on breast cancer data set and then compared these results with the results from original literature based on both somatic mutations and copy number alterations. Breast cancer data set were obtained from European Genome-Phenome Archive (accession EGAS00001000760) (Brown et al., 2017). The running time of PTI, as well as the other two methods, was short, just within seconds (**Table S5**). PTI and Treeomics both showed higher accuracy compared with LICHeE. In results predicted by PTI, 6 out of 8 patients showed identical structures, while the other two patients P1 and P2 showed highly similar tree structures as the results in Brown et al. (2017), which may be caused by more than one subclonal population in one biopsy (**Figure 3**). For example, in patient P1, sample M1 (Metastatic tumor sample) includes A-clone and B-clone, sample M4 (Metastatic tumor sample) contains only A-clone, and samples M3 and P contain B-clone. Therefore, the sample M1 is grouped together with the sample M4 or with the samples P-M3, which is determined by the proportion of somatic mutations involved in A-clone and B-clone in sample P (**Supplementary Note 6**). Treeomics also showed good performance, and 6 out of 8 results were identical. However, in the LICHeE results, 5 out of 8 patients showed single-branch or multi-branch differences in tree structures. We also tested these two methods on different AF cutoffs. The accuracy of the tree structures of the three methods was slightly improved, but PTI still showed better performance (**Table S6**). Moreover, we also demonstrated that PTI performed robustly in the low coverage data set by applying it to the HISEQ data set for 8 breast cancer patients (**Table S7**).

### Results on 13 Cancer Types Data Set

**Figure 4:**
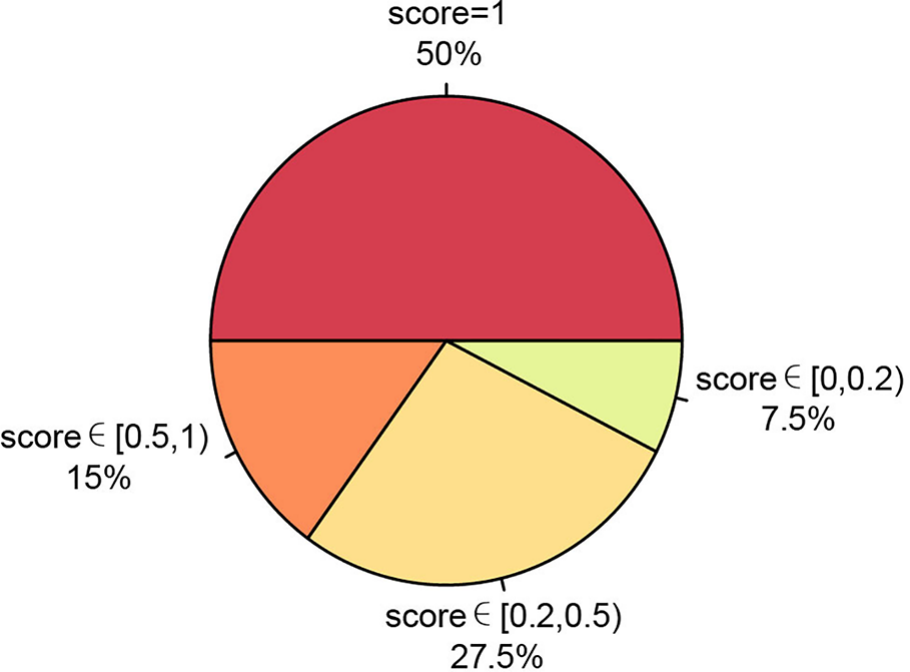
Summary of our method's performance on 13 cancer types data set without allele frequency. The consistency between PTI and original results based on similarity scores were represented as a pie chart.

We also ran PTI on a real data set including somatic single nucleotide mutations from 40 patients of 13 cancer types where allele frequency data is not available. This data set were obtained from BioStudies database (accession S-EPMC4776530) (Zhao et al., 2016). We applied PTI to infer the tree structure and then compared our results with the results that based on the multiple sequence alignments, maximum likelihood algorithm, the maximal parsimony algorithm, and the Bayesian inference criteria implemented in original study. And then, based on similarity score, we categorized the comparison results into four groups: similarity score = 1, similarity score∈ [0.5,1), similarity score ∈ [0.2,0.5) and similarity score ∈[0,0.2), representing various degrees of tree structure similarity. The comparison shows that 92.5% of our results have the same or similar tree structure (similarity score greater than 0.2) as the results in Zhao et al. (2016) (**Figure 4**, **Figure S8**), which again suggest that our method can be applicable in a wide range of applications.

## Discussion

As PTI assumes that there is one major clone in each biopsy, when there is more than one major subclone, PTI will assign the sample to the subclone with a higher mutation count by not their relative cellular abundances of two subclones (**Figure 3** and **Table S4**). This may lead to some discrepancies in the tree structures compared with other methods. But this case is rarely observed in the studies for multi-region sequencing, we only observe one case in all cases we tested.

In this study, we present PTI, a novel and easy-to-use method to infer the phylogenetic tree of tumor progression using just somatic mutations without the need for deep sequencing to obtain high-confident allele frequency measurement. Our comparison to other existing methods, such as LICHeE, Treeomics, BAMSE, and other traditional methods, shows that PTI achieves similar or slightly better performance within a short run time, normally less than a minute. This feature is important for studying clinical samples that are difficult to obtain accurate allele frequency information, such as formalin-fixed, paraffin-embedded (FFPE) samples. Moreover, the input file for PTI is a similar zero and one feature-by-sample matrix so that this method is generally applicable to infer phylogeny for any other data sets that can be converted into this format (such as epigenetics). In fact, this method is also well suited for single cell data sets to evaluate the similarity between single cells and construct their phylogenies.

## Data Availability Statement

Publicly available datasets were analyzed in this study. This data can be found here: The European Genome-phenome Archive (EGAS00001000547, EGAS00001000667, EGAS00001000760) and Biostudies (S-EPMC4776530).

## Author Contributions

PW and LZ designed the algorithm and interpreted the results. PW implemented the algorithm and conducted the analyses. LH assisted in obtaining and preprocessing the data sets. YZ assisted in setting up the software environment for performance comparison.

## Funding

This work is funded by the National Key Research and Development Program of China (2018YFC1004602), National Natural Science Foundation of China (NSF 31871332) and a startup fund to L.Z. from ShanghaiTech University.

## Conflict of Interest

The authors declare that the research was conducted in the absence of any commercial or financial relationships that could be construed as a potential conflict of interest.
