# Supplementary material for: Phylogenetic Tree Inference: A Top-Down Approach to Track Tumor Evolution
Source: Front Genet. 2020 Feb 7;10:1371. doi: 10.3389/fgene.2019.01371 (PMC7020887; doi:10.3389/fgene.2019.01371)
Supplement: Supplementary file 1 [file DataSheet_1.docx]

Supplementary Material

**Supplementary Note1: PTI can uncover more than two-way branch split structure**

PTI uses an iterative top-down approach to infer the phylogenetic tree for multiple samples of the same patient with only somatic mutations. Although more than two-way split can be rarely observed at a given evolutionary node which is caused by the complexity of cancer progression, our method indeed is able to detect more than two-way branch split. For example, in the phylogenetic tree structure of patient P429 from the 13 cancer types dataset, which is inferred by PTI, the second trunk level includes three-way branch split (**Figure S1**). In this case, PTI first generates the 2-3 split two-way branch split, and then the two samples are further split into two independent branches, as there are no shared mutations between them.


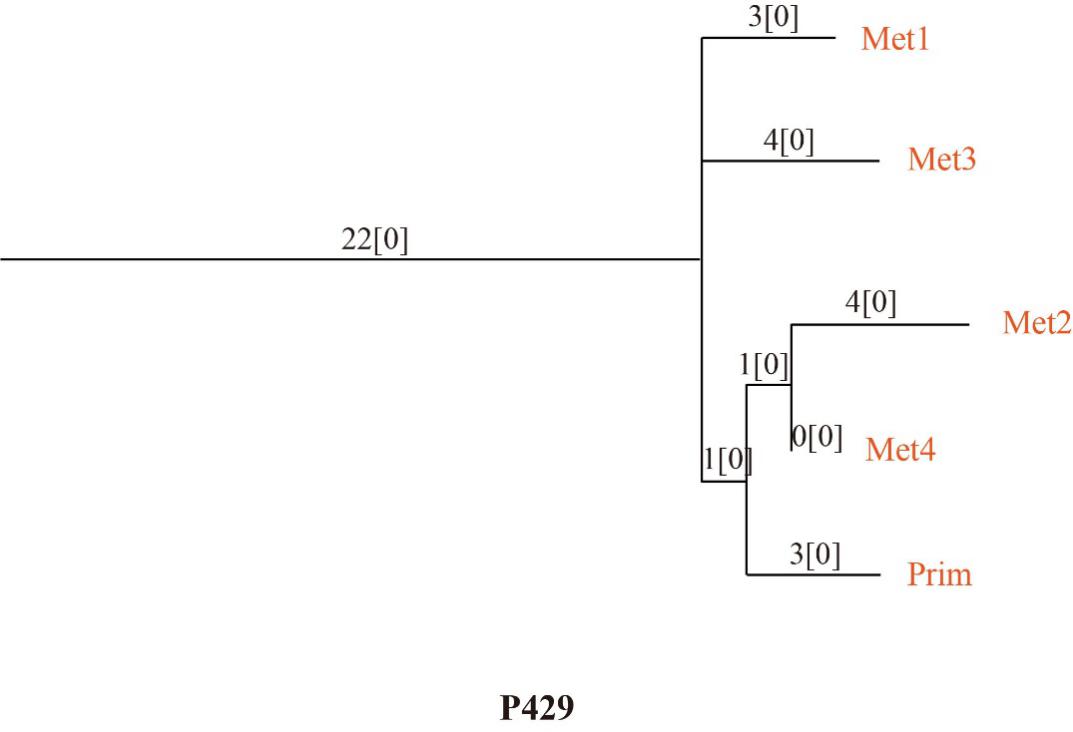


**Figure S1** The phylogenetic tree structure of patient P429 from the 13 cancer types dataset.

**Supplementary Note2: Rationale of using best and second best shared mutation count ratio to infer best branch split pattern**

In the process of finding the best branch split $\left( t,n-t \right)$ from all possible split patterns $\left\{ \left( 1,n-1 \right),\left( 2,n-2 \right),\ldots\left( \left| \frac{n}{2} \right|,n-|\frac{n}{2}| \right) \right\}$, we argue that the ratio between the best combination and second best combination should be much larger compared with non-optimal splits if the optimal split occurs in $S_{t}$. For example, from the phylogenetic tree structure of patient P400 from 13 cancer types dataset we can see that the optimal branch split has the maximum ratio, which is calculated by the maximum value and the secondary maximum value in $\theta_{t}$ (**Figure S2**).


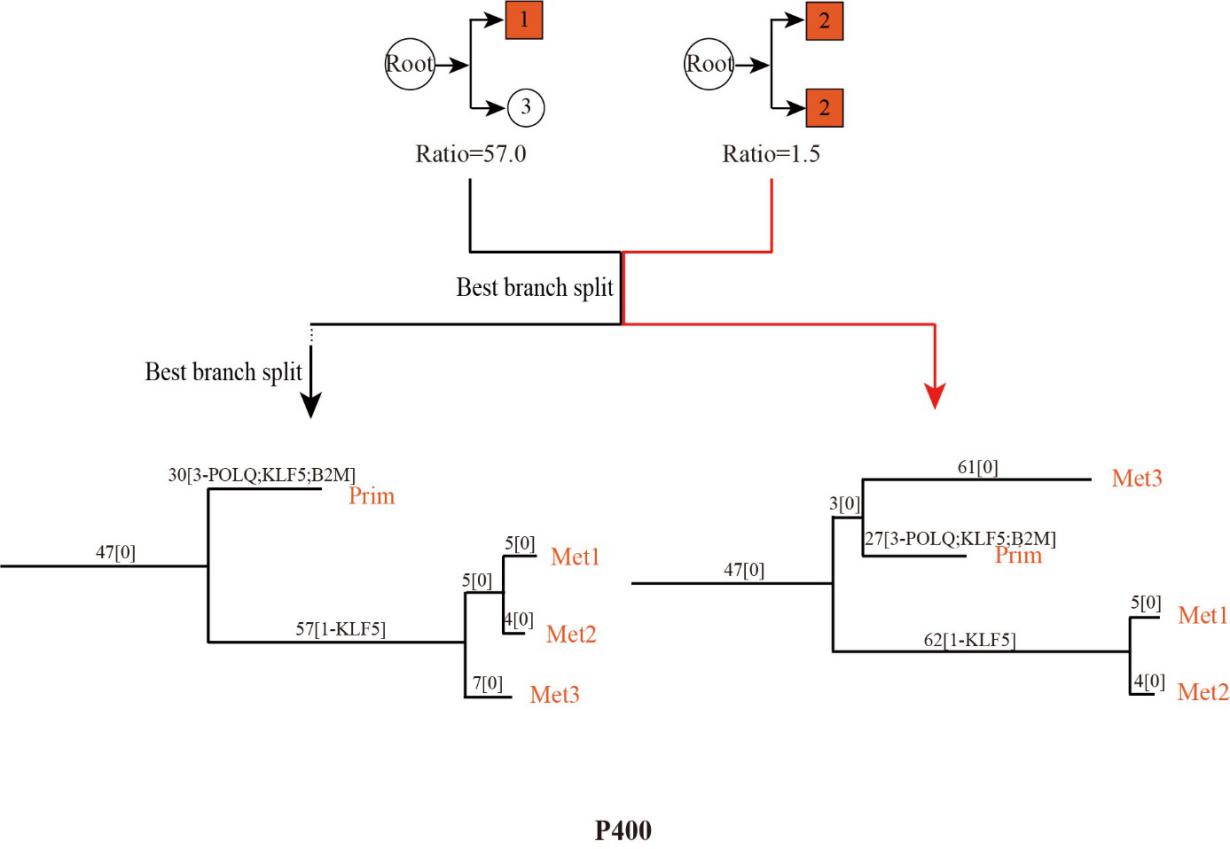


**Figure S2** The phylogenetic tree structure of patient P400 from the 13 cancer types dataset.

As for using the ratio of maximum number against the mean of all other splits, we have considered this option before. We have compared these two ratios to infer optimal tree structures on four cancer datasets. In fact, ratios of maximum number against the mean of all other splits often are equally good as the ratios of maximum vs second best. But in some cases (~6.5%), it does not function well to define the best branch in term of weight scores and similarity scores because the mean values in all branch split are approach zero infinitely (see detailed example in the following paragraph).

In the example from patient EV007 in ccRCC dataset, we can see that the first branch split inferred based on ${Max}/{\sec\_Max}$ ratio is correct based on consistent with gold standard as well as the weight score (**Table S1**).

**Table S1** The optimal first branch split inferred by PTI using ${Max}/{\sec\_Max}$ and ${Max}/{Mean}$ ratios in EV007.

| Patient: EV007 | Total samples: 10 If Mean<1, then Mean=1 | | | |
| --- | --- | --- | --- | --- |
|  | ${Max}/{\sec\_Max}$ | ${Max}/{Mean}$ | LICHeE | Published in original paper |
| As for first branch split | (3,7) split | (4,6) split | (3,7) split | (3,7) split |
| Weight score | 102 | 83 | NA | NA |
| Similarity score | 0.86 | 0.46 | 0.85 | NA |

To understand why in this case (and some other similar cases), ${Max}/{Mean}$ ratio leads to incorrect results. We examined this case in more details. We summarized the number of shared mutations in the bigger group for all possible splits and calculated the ${Max}/{\sec\_Max}$ and ${Max}/{Mean}$ ratios for each branch split (**Figure S3, Table S2**). In the **Table S2**, * indicate that when the number is less than 1 will be replaced as 1 in order to calculate the ratio.


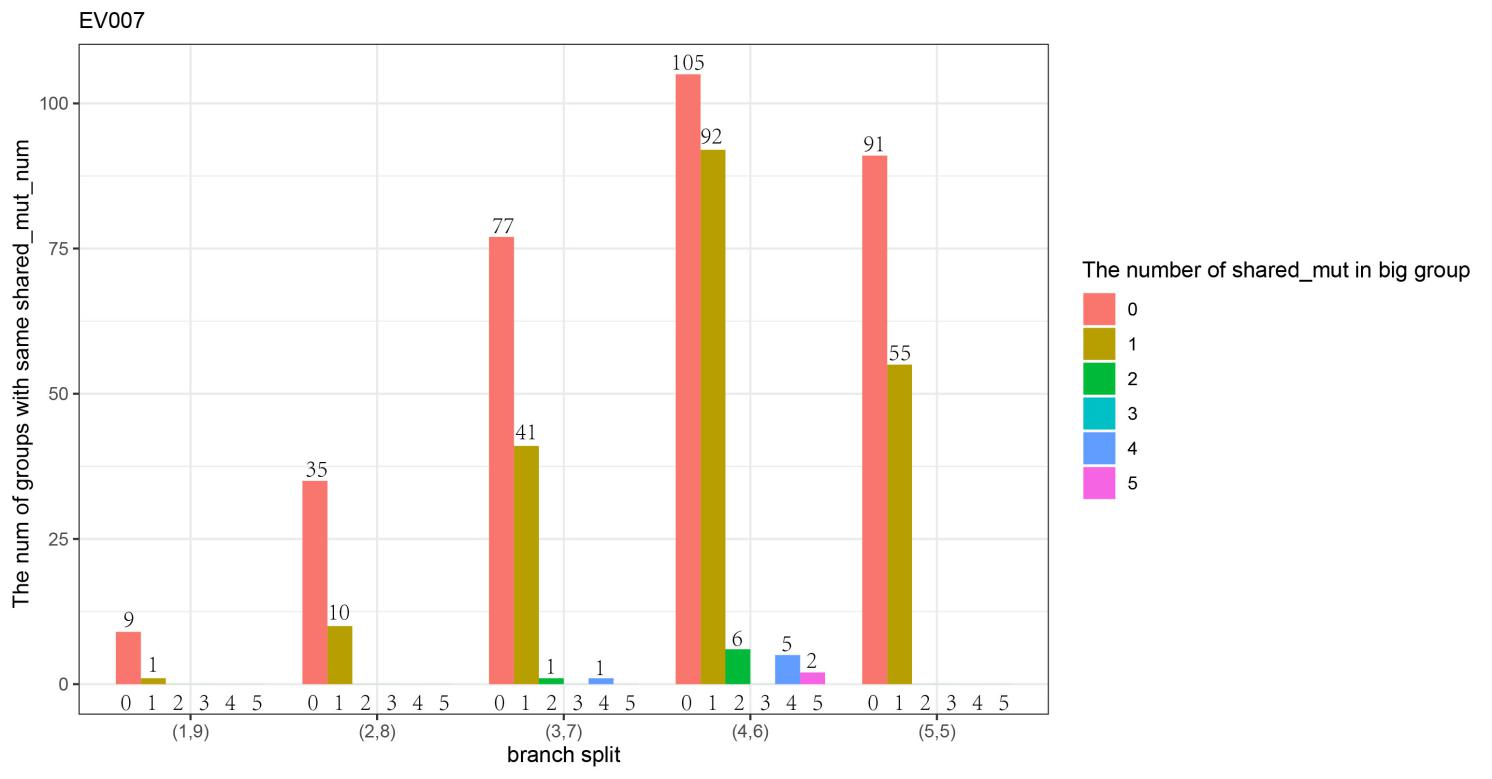


**Figure S3** The number of shared mutations in the bigger group for all possible branch splits in the first branch split in EV007.

**Table S2** The ${Max}/{\sec\_Max}$ and ${Max}/{Mean}$ ratios for all possible branch splits in the first branch split in EV007.

|  | (1,9) split | (2,8) split | (3,7) split | (4,6) split | (5,5) split |
| --- | --- | --- | --- | --- | --- |
| ${Max}/{\sec\_Max}$ | 1/1*=1 | 1/1=1 | 4/2=2 (select) | 5/5=1 | 1/1=1 |
| ${Max}/{Mean}$ | 1/1*=1 | 1/1*=1 | 4/1*=4 | 5/1*=5 (select) | 1/1*=1 |

**Table S3** The combinations with the most shared mutations in optimal first branch split inferred by ${Max}/{\sec\_Max}$ and ${Max}/{Mean}$ ratios in EV007.

| Split type | Sample split | Shared mutation count in the bigger group |
| --- | --- | --- |
| (3,7) split | [R3min,R4,R9dom] [**R1,R2,R3dom,R5,R6,R7,R9min**] | 4 |
| (4,6) split | [R3min,R4,R9dom,**R9min**] [**R1,R2,R3dom,R5,R6,R7**] | 5 |
| (4,6) split | [R3min,R4,R9dom,**R6**] [**R1,R2,R3dom,R5,R7,R9min**] | 5 |
| (4,6) split | [R3min,R4,R9dom,**R1**] [**R2,R3dom,R5,R6,R7,R9min**] | 4 |
| (4,6) split | [R3min,R4,R9dom,**R2**] [**R1,R3dom,R5,R6,R7,R9min**] | 4 |
| (4,6) split | [R3min,R4,R9dom,**R3dom**] [**R1,R2, R5,R6,R7,R9min**] | 4 |
| (4,6) split | [R3min,R4,R9dom,**R5**] [**R1,R2,R3dom,R6,R7,R9min**] | 4 |
| (4,6) split | [R3min,R4,R9dom,**R7**] [**R1,R2,R3dom,R5,R6,R9min**] | 4 |

Based on ${Max}/{\sec\_Max}$, (3,7) split is the best. We can also observed that only one split pattern generated the largest shared mutation count 4 in all the possible (3,7) splits (**Figure S3**), which is consistent with the assumption that only one split will be the best. In the (4,6) split, since the correct split is (3,7), any 6 samples out of the 7 samples from the correct branch split will yield higher shared mutation count numbers. In fact, 7 (4,6) splits showed shared mutation count of 4,5 are all from the 7 samples of correct (3,7) split (See table S3 above, sample from correct branch split are bolded and in red color). Therefore, ${Max}/{\sec\_Max}$ ratio in this case is equal to 5/5 yield a smaller number and will not be selected as the best split.

However, if we use mean value, in this case, all mean value is 1. And because the number of shared mutation will decrease when the group size is getting bigger, and mean value cannot control this effect, thus leading to an incorrect branch split. However, such effect will be adjusted if we are using ${Max}/{\sec\_Max}$. As you can see that the sec_Max counts keep increases until the equal split (from 1 to 2, then to 5).

To conclude, as we shown above sec_Max ratio serves as a better background value. Therefore, we believe ${Max}/{\sec\_Max}$ ratio is a better readout for optimal split compared with ${Max}/{Mean}$ ratio.

**Supplementary Note3: Cases with more than one equally optimal structure**

PTI will only output the phylogenetic tree with the largest weight score. In practice, we have found that most cases have only one optimal tree structure, but there are also a few cases where PTI will infer two or more similar tree structures for same patient with a single branch difference but have the same weight score, such as the phylogenetic tree structures of patient P446 from the 13 cancer types dataset. In these cases, PTI will report all the solutions with the highest scores. From the ancillary files of the phylogenetic tree we know that the production of multi-tree structures is caused by the presence of polyclone in M2 sample: M2 sample contains both clonal mutations that specific to M4 and M3.

**
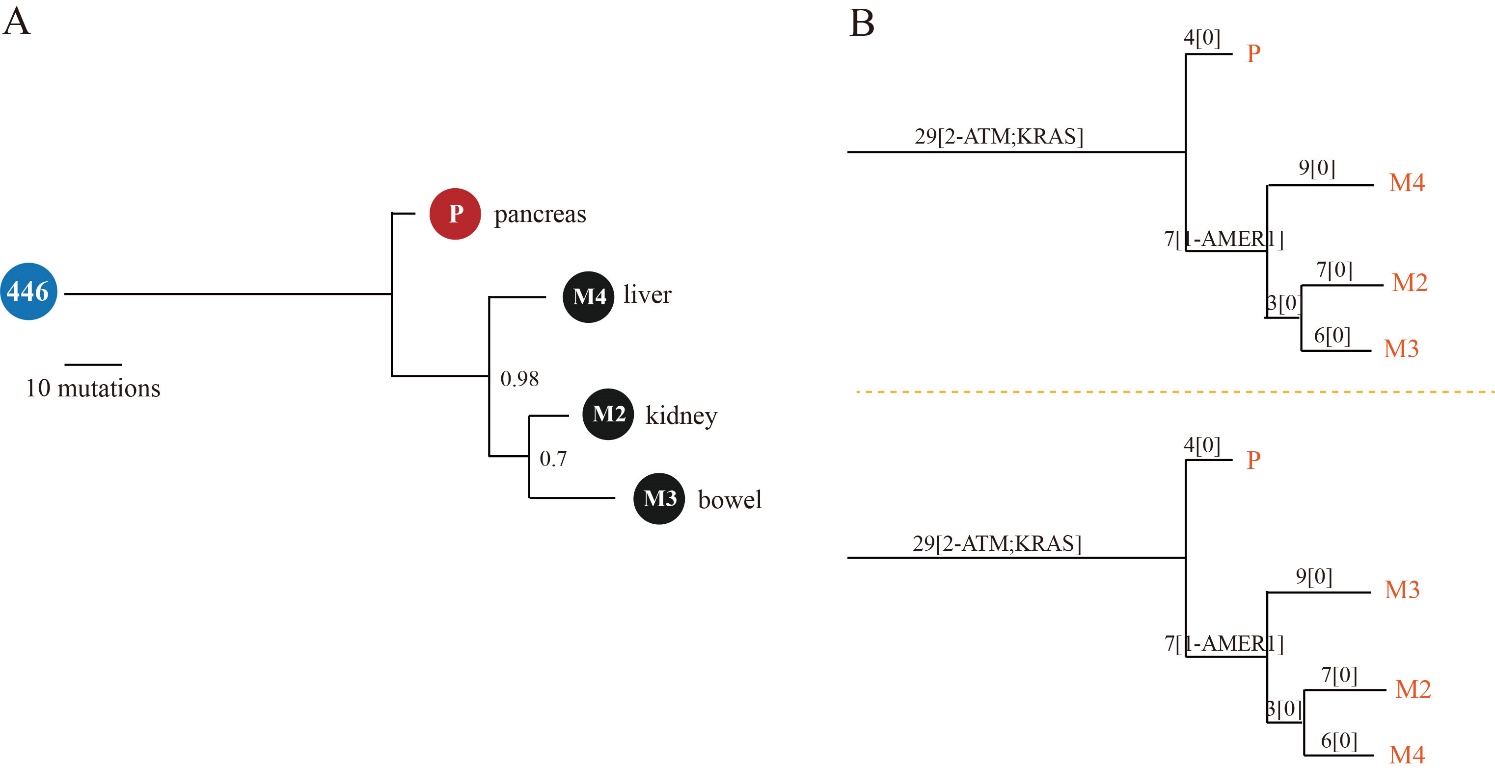
**

**Figure S4** An example of inferred tree structures for the patient P446. (**A**) The tree structure is given in the original published paper. (**B**) The tree structure is inferred by PTI. Topology of the tree on the upper right is the same as the one on the left. Both structures also show identical weight scores.

**Supplementary Note 4: tree structure similarity scoring system**

In order to evaluate the similarity of two tree structures, we defined a tree structure similarity scoring system. The similarity score represents the proportion of the same path in the tree topology and ranges from zero to one. The larger the score value, the more similar the tree structure is. The similarity score was calculated by the Equation:

$$Score=\frac{2*same\_path}{PATH1+PATH2}$$

in which PATH1 and PATH2 respectively represent all paths from two tree structures. Take patient Case3 in the High-grade serous ovarian cancer dataset as an example: the tree structure of Case3 given in the original paper has 5 paths in total, including (root, (a,b,c)), ((a,b,c), a), ((a,b,c), (b,c)), ((b,c), b) and ((b,c), c). As for the tree topology inferred by PTI, the number of all paths and the number of identical paths are 5. Using the Equation above, the similarity score is 1, which means that the two tree structures are identical.


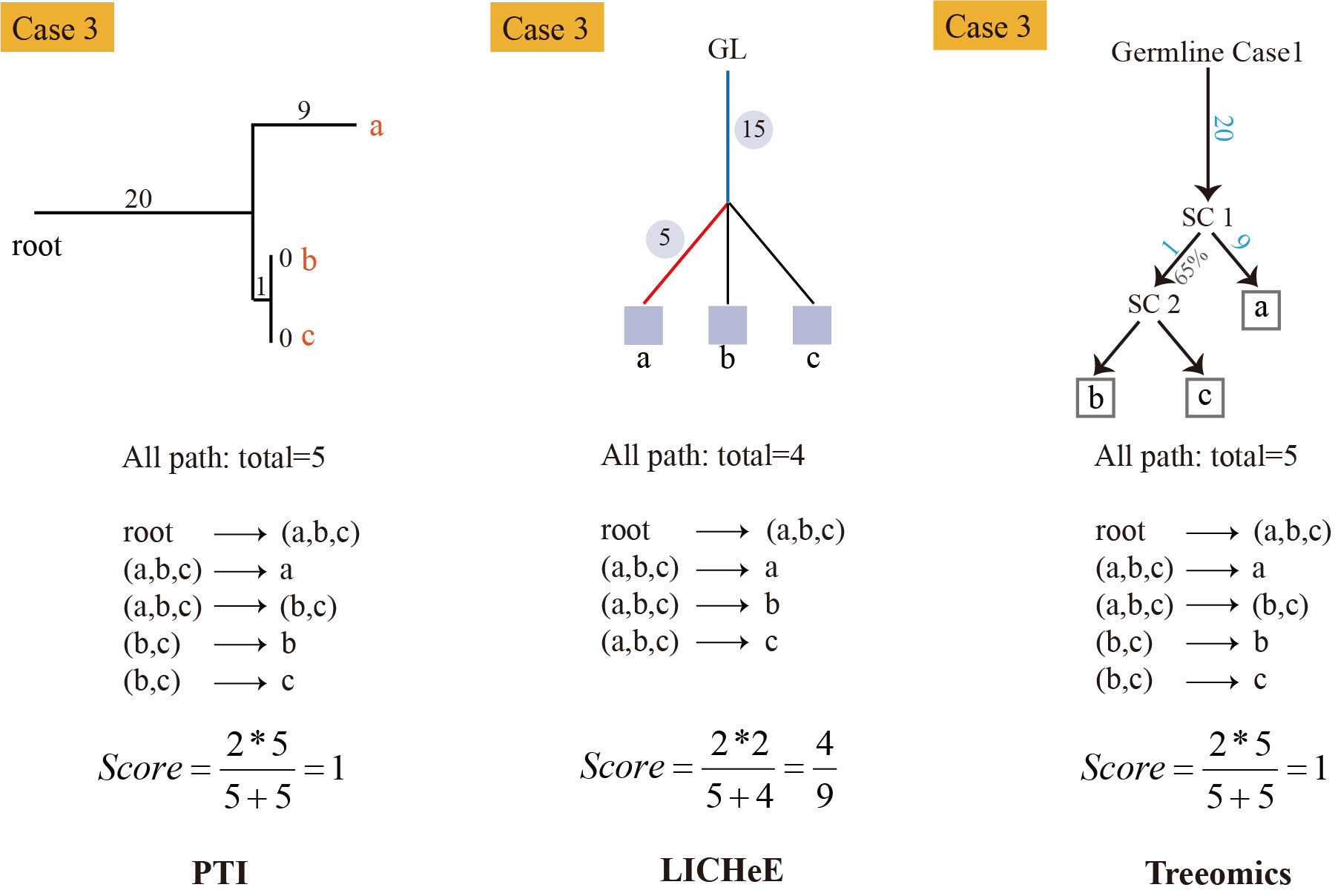


**Figure S5** The tree structure of Case3 in the HGSC dataset inferred by PTI, LICHeE and Treeomics.

**Supplementary Note 5: The tree structures of HGSC Case 1 and Case 5**

**
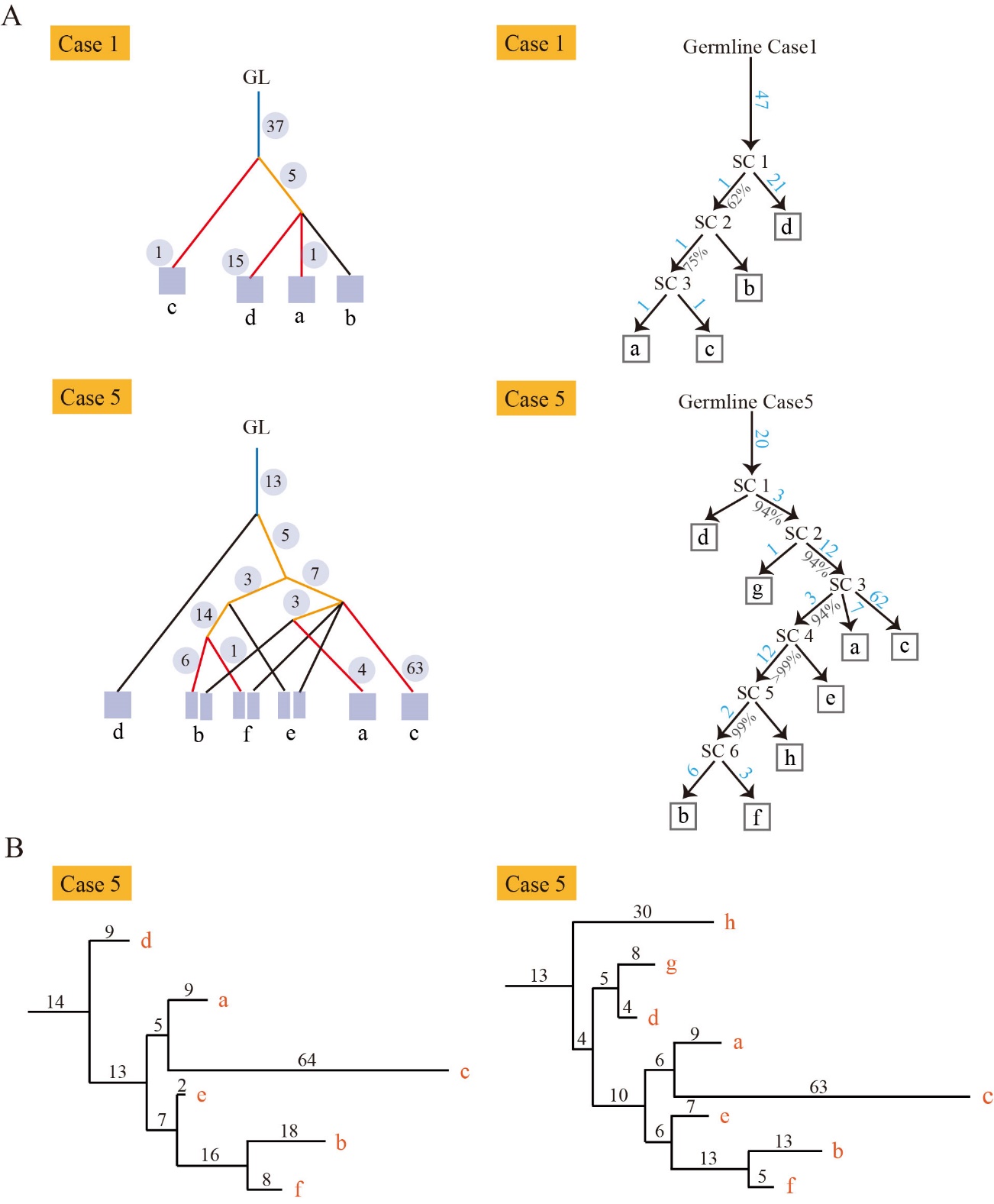
**

**Figure S6** The tree structures of HGSC Case 1 and Case 5. (**A**) The tree structure of Case 1 and Case 5 inferred by LICHeE (left) and Treeomics (right). In the LICHeE results: red line, terminal branch; yellow line, internal branch; light blue line, trunk; black line, contribution link; the numbers inside the circle, SSNVs; the light purple square, tumor region; split light purple square, mixed lineage. In the Treeomics result: numbers in blue correspond to the acquired variants in the branches. Percentages (gray) denote bootstrap values (1,000 samples). SC, subclone. (**B**) The tree structure of Case 5 inferred by PTI using six samples(left) and eight samples obtained from original paper with AF>=0.01 (right).

**Supplementary Note 6: Tree difference may be caused by more than one subclonal population in one biopsy**

As for breast cancer dataset, the patient P1 showed highly similar tree structure as the results in original paper, which may be caused by more than one subclonal population in one biopsy. For example, in patient P1, sample M1 (Metastatic tumor sample) includes A-clone and B-clone, sample M4 (Metastatic tumor sample) contains only A-clone, and samples M3 and P contain B-clone. Therefore, the sample M1 is grouped together with the sample M4 or with the samples P-M3, which is determined by the proportion of somatic mutations involved in A-clone and B-clone in sample P. In this case, in sample M1, A clones accounted for 75% and B clones accounted for 25%, so that M1 was more likely to be grouped together with the sample P and sample M3.

**Table S4** Subclonal mutations included in samples of patient P1.

| Clone | Somatic mutations (chromosome-position) |
| --- | --- |
| A-clone | 21-19670103 |
| B-clone | 2-182339901, 15-55483193, 19-38161022 |

**Supplementary Results:**


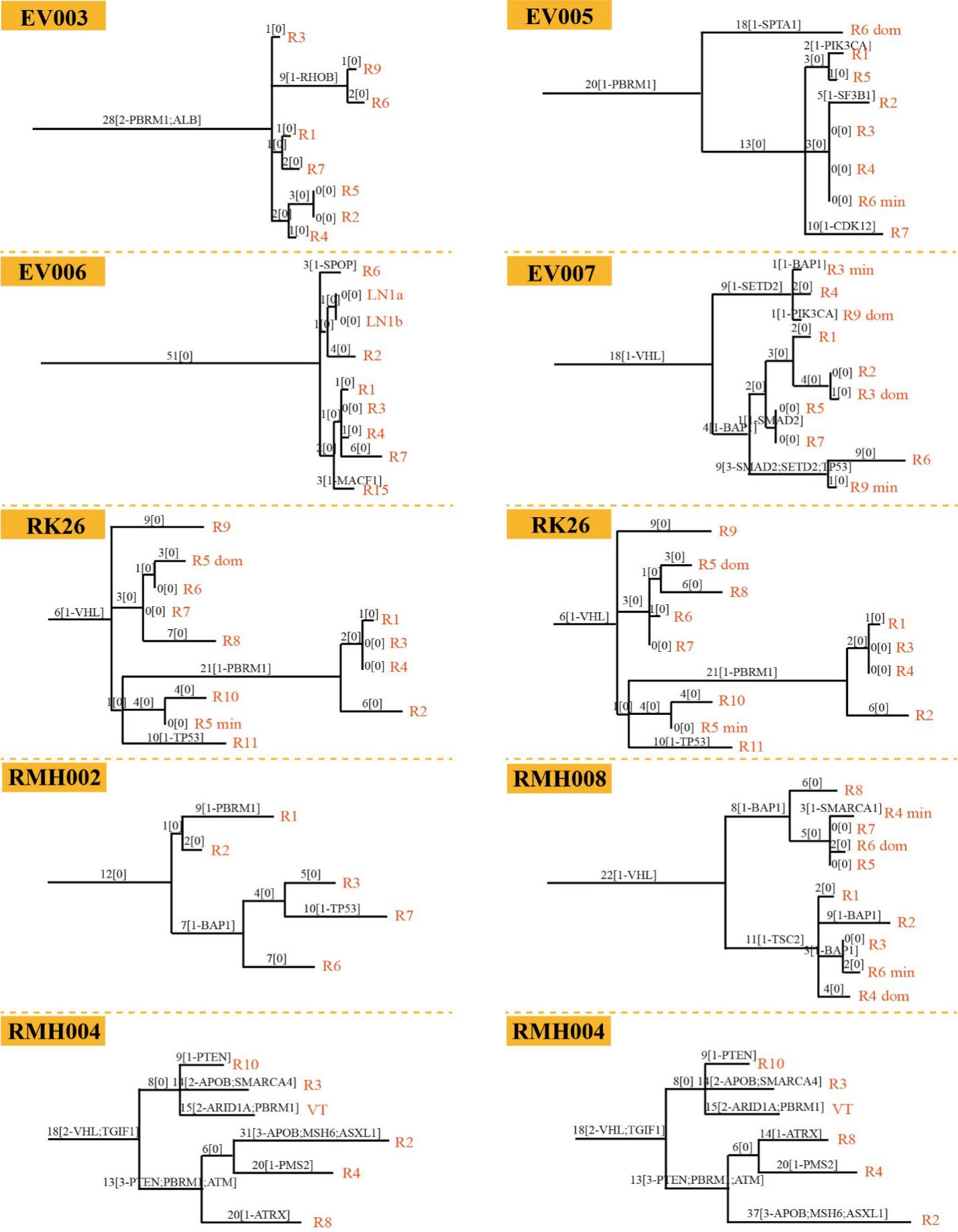


**Figure S7** All trees inferred by PTI based on ccRCC dataset. For patient RK26 and RMH004, PTI reports two solutions with equal weight score.

**Table S5** The running time of PTI and other state-of-the-art methods based on two dataset (CPU: AMD Ryzen 7 2700X Eight-Core Processor).

| Dataset | PTI | LICHeE | Treeomics |
| --- | --- | --- | --- |
| HISEQ in breast cancer  (10 patients, 65 samples, 1285 mutations) | 1.86 s | 16.78 s | 37.70 s |
| Deep-seq in breast cancer  (10 patients, 65 samples, 1040 mutations) | 1.97 s | 16.72s | 37.54 s |

**Table S6** The comparison of PTI and LICHeE based on breast cancer deep sequencing dataset.

|  | **PTI** | | | **LICHeE** | | | **Treeomics** |
| --- | --- | --- | --- | --- | --- | --- | --- |
| **Patient_ID** | **AF>=0.03** | **AF>=0.1** | **AF>=0.2** | **AF>=0.03** | **AF>=0.1** | **AF>=0.2** | **AF>=0.03** |
| P1 | 0.33 | 0.33 | 0.33 | 0.38 | 0.35 | 0.33 | 0.38 |
| P2 | 0.43 | 0.57 | 0.43 | 0.62 | 0.62 | 0.62 | 0.62 |
| P4 | 1 | 1 | 1 | 1 | 1 | 1 | 1 |
| P5 | 1 | 1 | 1 | 1 | 1 | 1 | 1 |
| P7 | 1 | 1 | 1 | 1 | 1 | 1 | 1 |
| P8 | 1 | 1 | 0.14 | 1 | 1 | 0.43 | 1 |
| P9 | 1 | 1 | 1 | 0.71 | 0.71 | 0.71 | 1 |
| P10 | 1 | 1 | 1 | 0.44 | 1 | 1 | 1 |

**Table S7** The comparison of PTI and LICHeE based on breast cancer low coverage sequencing dataset.

|  | **PTI** | | | **LICHeE** | | | **Treeomics** |
| --- | --- | --- | --- | --- | --- | --- | --- |
| **Patient_ID** | **AF>=0.03** | **AF>=0.1** | **AF>=0.2** | **AF>=0.03** | **AF>=0.1** | **AF>=0.2** | **AF>=0.03** |
| P1 | 0.2 | 0.2 | 0.2 | 0.2 | 0.2 | 0.2 | 0.2 |
| P2 | 0.14 | 0.14 | 0.14 | 0.14 | 0.14 | 0.14 | 0.14 |
| P4 | 1 | 1 | 1 | 1 | 1 | 1 | 1 |
| P5 | 1 | 1 | 1 | 0.93 | 1 | 1 | 1 |
| P7 | 1 | 1 | 1 | 1 | 1 | 1 | 1 |
| P8 | 1 | 1 | 1 | 0.83 | 1 | 0.44 | 1 |
| P9 | 1 | 1 | 1 | 0.71 | 1 | 0.56 | 0.56 |
| P10 | 1 | 1 | 1 | 1 | 1 | 1 | 1 |

**
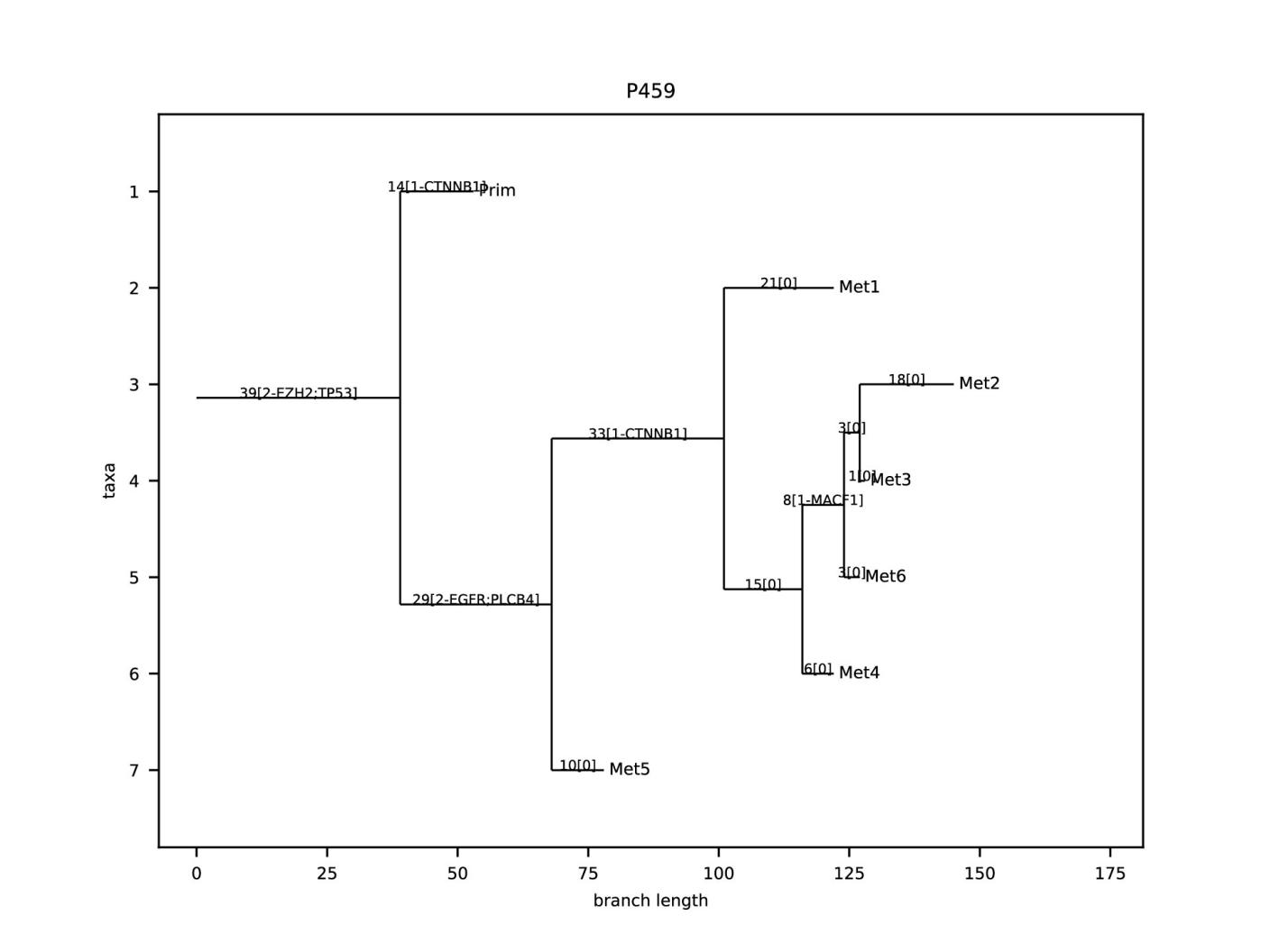
**

**Figure S8** An example of trees inferred by PTI based on 13 cancer types dataset. All tree structures can be found in https://github.com/bioliyezhang/PTI.
